# A novel strategy to purify conductive polymer particles†

**DOI:** 10.1039/c8ra08372j

**Published:** 2019-02-08

**Authors:** Daron Spence, Georgios Polizos, Pooran Joshi, Jaswinder Sharma

**Affiliations:** Roll-to-Roll Manufacturing Group, Oak Ridge National Laboratory Oak Ridge TN 37831 USA sharmajk@ornl.gov +1-865-241-2333; Building Envelope & Urban Systems Research Group, Oak Ridge National Laboratory Oak Ridge TN 37831 USA; Material Science and Technology Division, Oak Ridge National Laboratory Oak Ridge TN 37831 USA; Georgia Institute of Technology Atlanta GA 30332 USA

## Abstract

The purification of conductive polymer (*e.g.*, polyaniline) particles is a challenging task, especially when the particle size is small. Herein, we demonstrate a unique strategy (electrode-based) to purify polyaniline particles by exploiting the difference in surface charge between particles and surfactants, and compare the results with a commonly used purification strategy (washing).

## Abstract 2

A novel strategy to purify conductive polymer nanoparticles is presented.

## Graphical abstract

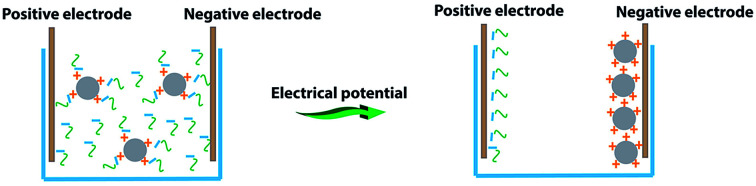

Conductive polymer (*e.g.*, polyaniline; PAni) particles are highly useful for applications such as the sensing of gases, humidity, or biological molecules, and making conductive composites and energy storage devices.^1–13^ Several of these applications exploit the electrical properties of these particles. For example, in ammonia sensors, the sensor material (PAni) has higher electrical conductivity in the absence of ammonia vapors. The interaction of ammonia vapors with PAni reduces its electrical conductivity, which in turn is converted into a readable digital signal.^1^ The electrical properties of PAni particles depend mainly upon the oxidation state of the PAni.^14,15^ The fully oxidized state of PAni is an electrical insulator, but the partially oxidized state is conductive. The conductivity peaks when approximately 50% of the nitrogen atoms in the PAni backbone are oxidized.^11^ The electrical conductivity of PAni particles also depends upon the degree of crystallinity of the particles, *i.e.*, the higher the crystallinity, the higher the electrical conductivity.^11,12^ PAni particles are generally synthesized by one of two approaches: (1) electrochemical polymerization or (2) chemical polymerization of aniline.^1,2,15–18^ Electrochemical synthesis consists of the formation and polymerization of free radical cations of aniline monomers into crosslinked PAni chains (particles) by employing electrical potential. Electrochemically made particles do not require much purification as no surfactants are present in the reaction mixture, however, resulting particles are less crystalline and less conductive.^11^ Chemical synthesis involves the use of polymerization agents such as potassium persulfate (KPS) or ammonium persulfate (APS) in the presence of surfactants such as sodium dodecyl sulfate (SDS), sodium dodecylbenzene sulfonate (SDBS), and dodecylbenzenesulfonic acid (DBSA).^1,2^ Generally, chemically synthesized PAni particles possess better crystallinity and electrical conductivity compared to the particles synthesized electrochemically. Additionally, chemical synthesis can provide particles at large scale and is suitable for industrial scale production. Therefore, chemical synthesis of PAni particles is the approach of choice.

Negatively charged surfactant ions dodecylbenzene sulfonate or dodecyl sulfate stick to the positively charged PAni particles. Surfactants help in controlling the particle size during synthesis and increasing the stability of particle suspensions after synthesis. A small number of surfactants sticking to the particles also helps in increasing the electrical conductivity by doping the PAni backbone.^18–20^ Though, surfactants, sticking to the particles, are helpful, free surfactants in the reaction mixture can lower the overall electrical conductivity of the particle mixture as surfactants themselves have very low electrical conductivities (≈6.0 × 10^−6^ S cm^−1^, measured by us on a DBSA film). Therefore, removal of extra surfactants from the reaction mixture is required in order to achieve particles with better electrical conductivity.

There are two general strategies to remove the extra surfactants from the particle samples: (1) centrifugation-based washing and (2) dialysis. Centrifugation-based washing is an inefficient method because particles are electrically charged and are typically small (<100 nm), thus require higher centrifugal forces to separate from the surfactants. It needs several cycles of centrifugation followed by removal of supernatant, addition of new solvent (generally ethanol or water) and repetition of centrifugation–washing cycles. Therefore, centrifugation-based washing is a tedious process, which wastes large amount of solvent and even after several washing cycles, the particles still contain an excess of surfactants.^2^ Another strategy, the use of dialysis membranes, has also been explored to separate the particles from surfactants.^1^ In this strategy, the particle sample containing excess surfactants is poured in a cavity surrounded by a dialysis membrane, then the cavity is submerged in large amount of pure water or water containing a certain amount of surfactants. Concentration gradients drive small sized surfactants to pass through the membrane, while the larger solid particles remain within the membrane walls. However, this technique takes several days and still does not provide pure particles. Therefore, purification of conductive polymer (*e.g.*, PAni) particles still needs more innovative strategies.

In the present work, we combined chemical synthesis with electrode-based purification to obtain high quality and highly pure particles. To achieve the higher crystallinity, controlled size, and better electrical conductivity, particles were synthesized by using chemical synthesis. To achieve the higher purification of particles, the particles were extracted by using electrode-based separation where positively charged particles moved towards negative electrode and negatively charged surfactants moved towards positive electrode.

PAni particles were prepared by employing a reported method.^1,2^ Briefly, 3.4 g of DBSA was dissolved in 30 mL of deionized water. Then, 0.6 mL of aniline was added to the DBSA–water solution. Next 0.36 g of ammonium persulfate (APS) was dissolved in 10 mL of deionized water and added to the aniline–DBSA–water mixture. The reaction mixture was stirred for 3 h, and a rusty colored viscous solution was obtained, which indicated the formation of PAni particles. Next, the as prepared solution of PAni particles was diluted with a water (50%)–ethanol (50%) solution. The reaction mixture had a pH ≈ 3.2 (quite below of PAni isoelectric point ≈ 8.2).^21,22^ This indicates that most of the PAni particles were in a positively charged state. Then two metal (copper) strips, one attached to the positive electrode and the other to the negative electrode of a power supply, were inserted in the diluted particle solution. A running experimental setup is shown in Fig. 1a. We found that at voltages lower than 0.3 V, the particle collection process was very slow while at higher voltages (≥1 V), particles aggregate irreversibly on the copper strip. The optimized voltage, used in all our experiments, was 0.6 V.

**Fig. 1:**
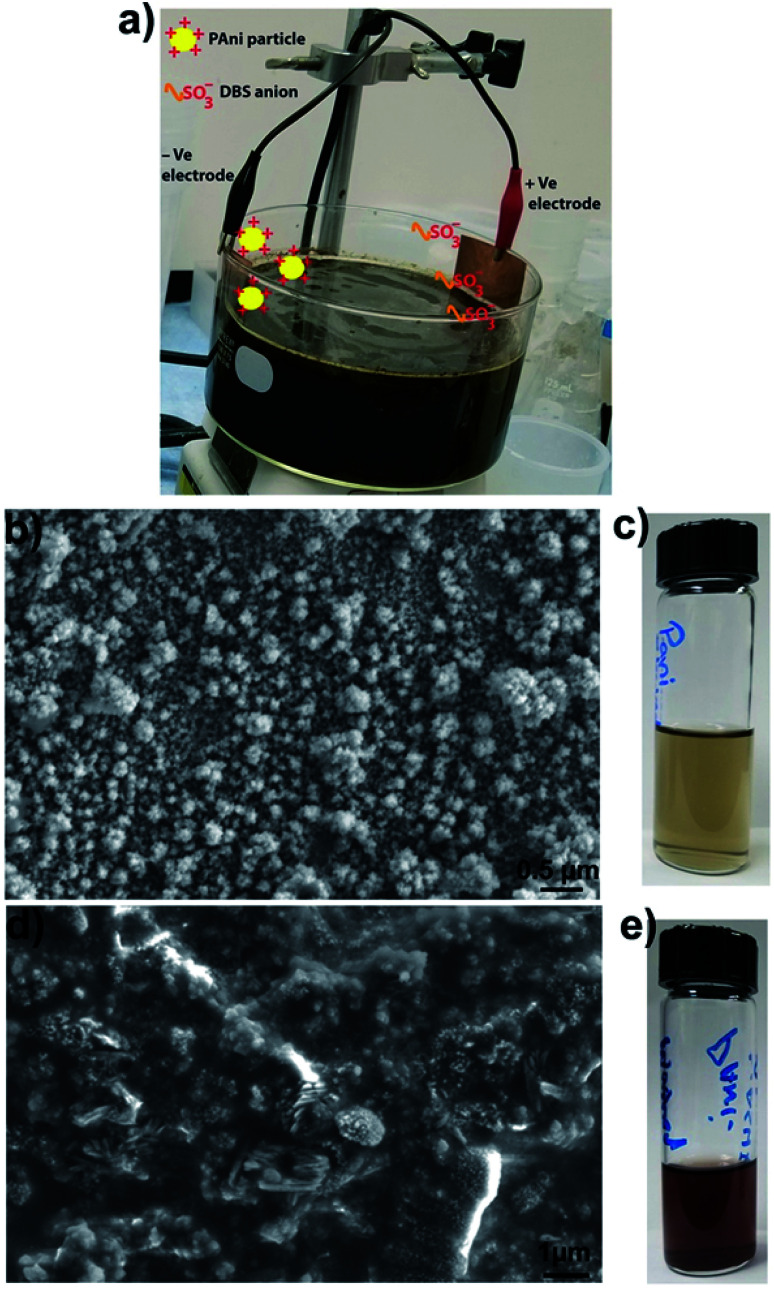
(a) Schematic and experimental setup used to purify the particles where red cable is positive electrode and black cable is the negative electrode, (b) SEM image of the particles purified by electrode-based method, (c) photo of particle solution obtained after electrode-based purification, (d) SEM image of particles purified by centrifugation–washing cycles, and (e) particle solution obtained after centrifugation—washing based purification.

Positively charged PAni particles start collecting on the copper strip attached to the negative electrode and negatively charged surfactants (dodecylbenzene sulfonate ions) start collecting on the copper strip connected to the positive electrode. Fig. 1b shows the scanning electron microscope (SEM) image of loosely bounded PAni particles collected on the copper strip connected to the negative electrode. SEM imaging showed that individual PAni particles were polydisperse in nature and have a size range ≈40–100 nm. PAni particles that seem larger than 100 nm are loosely bounded clusters of smaller particles, which can be easily dispersed in ethanol–water mixture.

After collecting the particles on the copper strip for 30 minutes, the metal strip containing the PAni particles was dipped in an ethanol–water (50 : 50 volume%) solution and sonicated for 10 minutes to strip off and disperse the particles. This process was repeated several times to increase the concentration of the particles in the solution. The amount of particles collected depends upon the surface area of the copper strip connected to the negative electrode of the power supply, *i.e.*, the larger strip collects more particles from the solution mixture. Fig. 1c shows the PAni particles stripped off the copper strip in an ethanol–water mixture. Fig. 1d shows the SEM image of PAni particles obtained after four cycles of centrifuging–washing with ethanol–water mixture and Fig. 1e shows the photo of the same particle suspension. SEM image of washed particles shows that substantial debris (surfactants) still remains in the particle solution and it is difficult to get rid of surfactants completely. Comparing the SEM image of particles obtained by electrode-based purification (Fig. 1b) with the SEM image of particles obtained by washing (Fig. 1d), it becomes clear that particles obtained by electrode-based purification are more purified than those obtained by centrifugation-based washing. Additionally, the comparison of the color of particle suspensions (Fig. 1c and e) also shows that particles obtained by washings have a rusty color very similar to that of the as synthesized particle solution (Fig. 1a), while particles purified by electrode separation are clearer and greenish in color. This further indicates that electrode-based purification provides cleaner particles than those were obtained by washings.

To check if any DBSA molecules are sticking on the particles purified by electrode-based method, energy dispersive X-rays (EDX) analysis was performed on the samples using a Merlin scanning electron microscope fitted with EDX capabilities. Fig. 2 shows the element maps and element percentages of copper strip alone and a copper strip having PAni particles on it. Element percentages were generated under automatic setting of the instrument. EDX elemental map and elemental percentages of copper strip alone did not show presence of any sulfur atoms (sulfonic group of DBSA). However, EDX elemental map and elemental percentages of copper strip with particles on it showed presence of a small amount of sulfur atoms (sulfonic group of DBSA), which indicates that some DBSA molecules are still sticking to the PAni particles. This shows that trace amounts of DBSA molecules don't detach from the particles under applied voltages. Additionally, DBSA molecules that may have been incorporated inside the particles during polymerization remained attached to the particles. We deduce that electrode-based purification mainly removes the excess surfactants present in the reaction mixture while surfactants attached firmly to the particle surface or incorporated inside the particle get collected at the negative electrode.

**Fig. 2:**
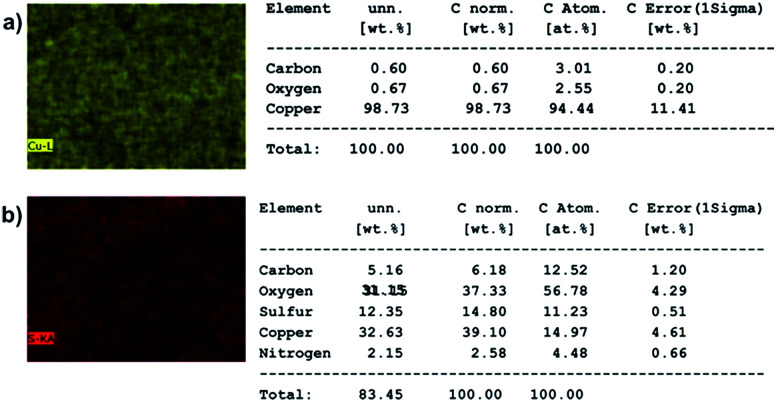
EDX studies showing presence of a small amount of sulfur (from sulfonic group of DBSA) molecules in particles purified by electrode-based purification. (a) EDX element map (left) and element percentages (right) of alone copper strip (b) EDX element map (left) and element percentages (right) of copper strip having PAni particles on it. Here, unn.: un-normalized; C. norm: corrected normalized; C. atom: corrected atomic, C. error: corrected error.

To further confirm that electrode-based particles are more purified than those obtained from washing–centrifugation approach, we performed FTIR (Fourier transform infrared spectroscopy) analysis (spectra shown in Fig. S7, ESI†). FTIR spectra of electrode-based particles showed sharp peaks compared to the spectrum obtained from washed particles. For example, characteristic peak for N–H stretching vibration of amino group of polyaniline at 3741 cm^−1^ is clearly visible in electrode-purified particles while it is buried in free DBSA molecules in the washed sample.^23^ Similarly, small peak at 2350 cm^−1^ (N–H)^+^ vibration related to unsaturated amine is missing in washed sample.^23^

We measured the electrical conductivity of particles purified by using electrode method and compared it with those of unwashed and centrifugation-based washed particles. To measure the electrical conductivity, we made thin 0.3 mm films of the particles on glass slides and allowed them to dry. A Jandel four-point-probe-device was used to measure the electrical sheet resistance of the particle films deposited on the glass slides. The measured sheet resistance values were used to calculate the resistivity and conductivity of the films. Fig. 3 shows a plot of electrical conductivity of as synthesized unwashed particles, particles purified by conventional centrifugation-based washings, and particles purified by electrode-based separation. Inset photos show the respective glass slides used for electrical conductivity measurements. The electrical conductivity (3.00 × 10^−4^ S cm^−1^) of the particles obtained by electrode purification was ≈20% higher than those of obtained by washing (2.50 × 10^−4^ S cm^−1^). Very low electrical conductivity (1.44 × 10^−4^ S cm^−1^) of unwashed (as synthesized) particles indicates that excess (free) surfactants (DBSA) are detrimental to the electrical properties of the PAni particles, and some kind of purification is always required to achieve the better electrical properties.

**Fig. 3:**
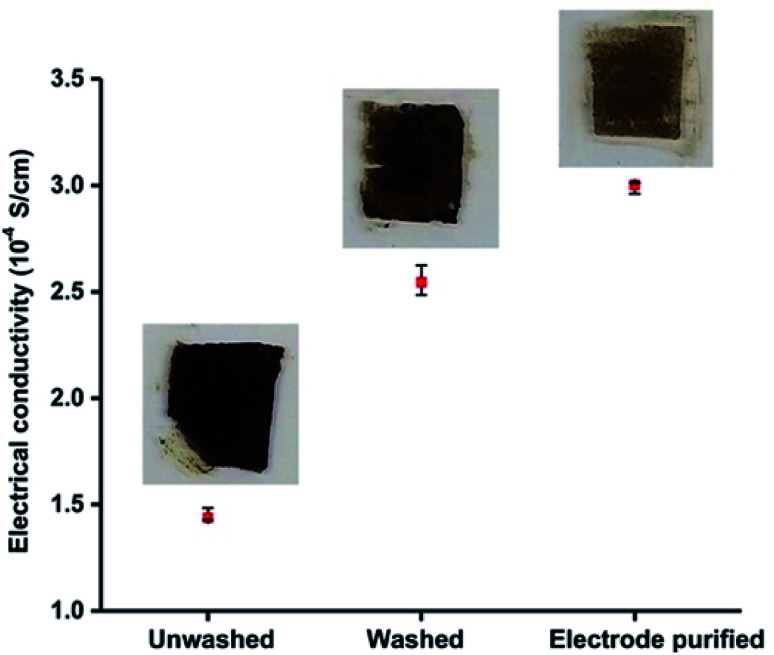
A graph showing the electrical conductivity of unwashed, washed, and particles purified by electrode-based separation. Insets: photographs of three samples used for measuring electrical conductivity.

Particles purified by electrode-based separation start aggregating into large lumps of PAni particles over time. Fig. 4 shows the photos and SEM images of particle suspension taken at different time intervals. As can be seen from the photos, the suspensions are clear in the early stages and start becoming opaque with time and finally particles start settling down at the bottom of the vial (Fig. 4a–d; left). SEM images (Fig. 4a–d; right) show a corresponding increase in particle aggregation. We measured the UV-Vis absorption spectra of the particle suspension at different time intervals, which showed that absorption at wavelength ≈420 nm (polaron band)^24^ keeps on increasing with time and finally stabilizes (Fig. 4e). We assume that this increase in absorption at 420 nm results from the aggregation of PAni particles with a corresponding loss of surfactants from particle surfaces.

**Fig. 4:**
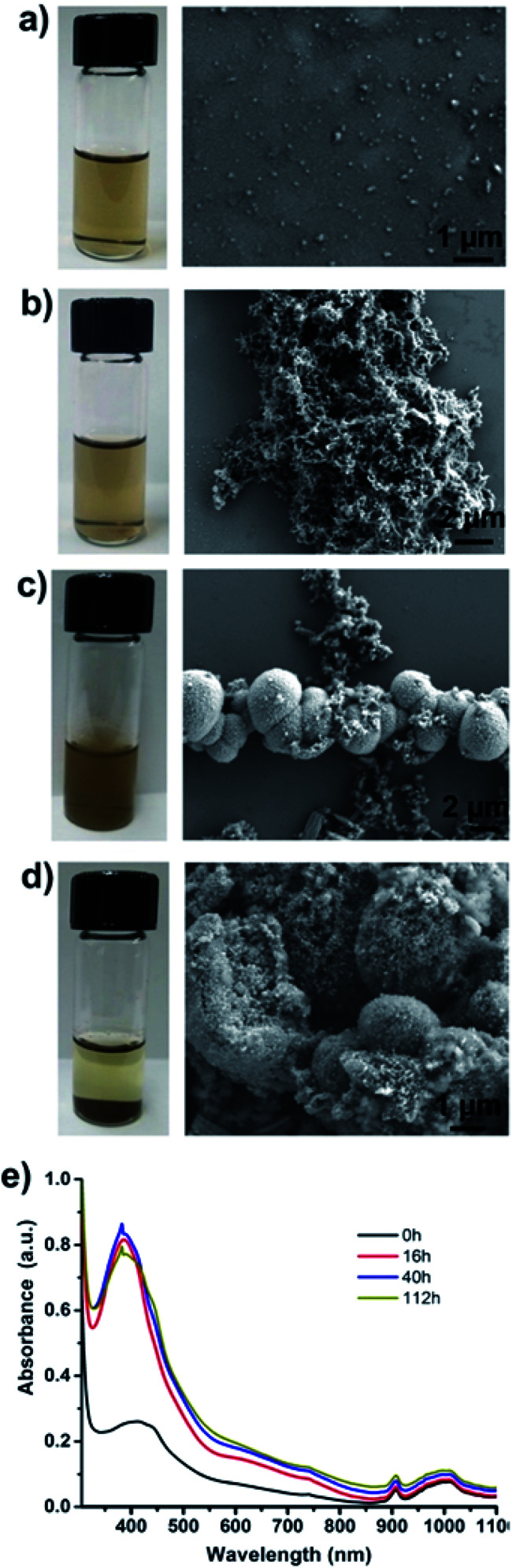
Aggregation of PAni particles separated by electrode method with time. Photos of PAni particles stripped of the electrode (left) and SEM image of the same sample (right). (a) 0 h, (b) 16 h, (c) 40 h, and (d) 112 h after striping the particles of the electrode. (e) Corresponding UV spectra of above samples. Note: UV spectra of sample at 112 h sample is measured after shaking the suspension.

Aggregation and phase separation of PAni particles in polar (hydrophilic) solvents is understandable when there is minimal amount of free (excess) surfactants in the solution as in our case. Initially, surfactants are bound to the PAni particles through their hydrophilic head while hydrophobic tail remains suspended in the solution. Over time surfactant molecules start aggregating or rearranging to make micelles allowing PAni particles free of DBSA to start aggregating with each other due to hydrophobic–hydrophobic interactions. Fig. S6 (ESI†) provides the assumed mechanism of PAni particle aggregation. However, if desired, addition of a small amount (30–35 μL of as obtained DBSA solution from Sigma Aldrich to 20 mL of particle solution) of surfactant helps in keeping the particles stable for about a week. The amount of surfactant to be added can be calculated by estimating the number of particles in the solution in order to avoid excess of surfactants in the particle sample. The aggregated particles can be redispersed by sonication or by adding a small amount of extra DBSA followed by shaking or sonication.

## Conclusions

We combined chemical synthesis with electrode-based purification in order to achieve highly purified PAni particles. The particles obtained by electrode-based purification show higher electrical conductivity when compared to the same particles but purified by using centrifugation-based washings. We found that if voltage is high (≥1.0 V) during separation process, it can result in aggregation and irreversible sticking of PAni particles on the surface of electrode, which results in poor extraction and dispersion of particles. Optimized voltage was ≈0.6 V, which separates the particles while avoiding their aggregation and irreversible sticking on the electrode surface. We anticipate that this work will further lead to purification of other conductive polymer particles or particle mixtures where conventional purification approaches are either not possible, time consuming, or expensive.

## Conflicts of interest

There are no conflicts to declare.

## Supplementary Material

RA-009-C8RA08372J-s001
