# Supplementary material for: A novel strategy to purify conductive polymer particles
Source: RSC Adv. 2019 Feb 8;9(9):4857–61. doi: 10.1039/c8ra08372j (PMC9060582; doi:10.1039/c8ra08372j)
Supplement: RA-009-C8RA08372J-s001 [file RA-009-C8RA08372J-s001.pdf]

Electronic Supplementary Material (ESI) for RSC Advances.
This journal is © The Royal Society of Chemistry 2019

Electronic Supplementary Information
A novel strategy to purify conductive polymer particles

Chemicals: Sodium dodecylbenzensulfonic acid (DBSA), aniline, and ammonium persulfate were purchased from Sigma-Aldrich.

Particle synthesis and purification: Particle synthesis was carried out by adding the ammonium persulfate solution dropwise into the solution of aniline and DBSA. The reaction mixture was stirred vigorously at 700 rpm for 3 hours. The as synthesized particle solution was diluted with a solution of 50 percent di water and 50 percent ethanol.

Particles purified by centrifugation-based washing: Purification of the particles was done through four cycles of centrifugation washing with ethanol- water (50:50) solution.
Particles purified by electrode method: were harvested by sonicating the copper electrode strip in an ethanol-water (50:50) solution.

All particles samples (unwashed, washed, and electrode based) were allowed to concentrate for 6 hours in a drier at 55 ^{O} C before the fluids were used to cast PAni films (≈0.3 mm thick) on glass slides.

Characterization: A Merlin scanning electron microscope was used to observe the surface structure of the films and ensure they were continuous. Electrical film resistance was measured using a Jandel 4-point probe and converted to electrical resistivity by multiplying by the height of the films. Then the inverse of the resistivity was taken to calculate the conductivity. The height of the sample was within 30 percent of the spacing between needles therefore no correction factor was employed.

ATR-FTIR : Washed PANI particles and electrode extracted were characterized via ATR-FTIR in a Bruker Vertex 70 FTIR using a vertical ATR apparatus at resolution 4 cm^{-1}. Samples were deposited onto a silicon crystal wafer for analysis. For each sample, 200 background scans on the wafer without sample were collected, then the sample was deposited, and a final spectrum of 100 averaged sample scans was produced.

PANI particles : Low voltage separation

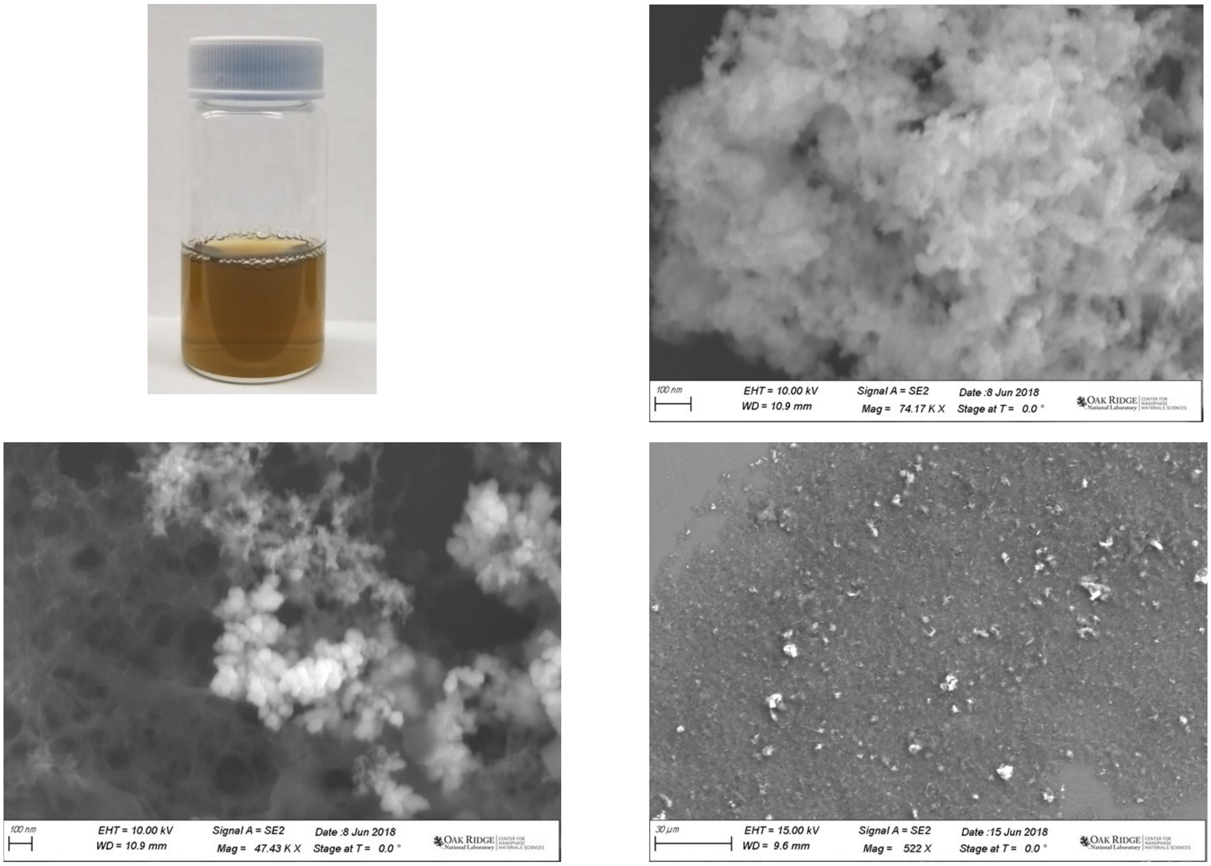

Figure S1. Showing PAni particle dispersion in ethanol-water (50%;50%) stripped of the electrode (upper left) and SEM images of the same when particles are separated by using a voltage ≈0.6 V.

PANI particles: High voltage separation

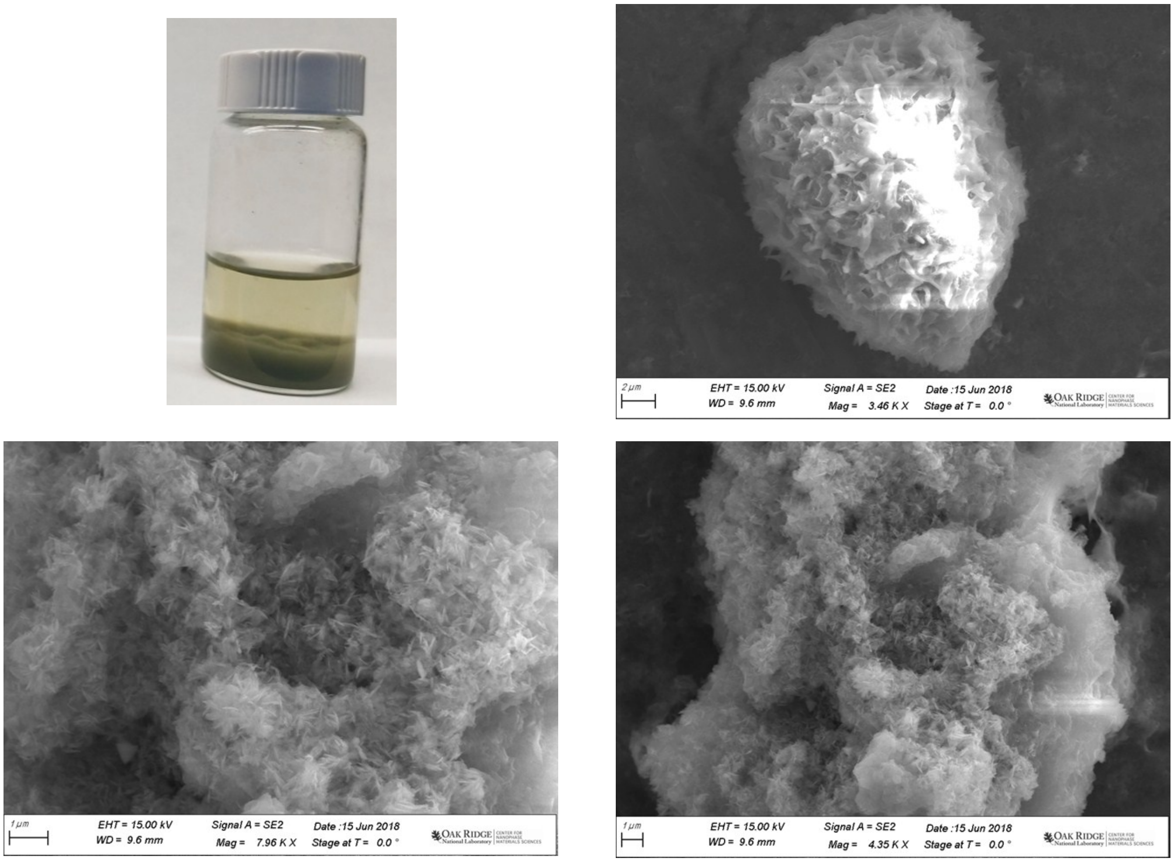

Figure S2. Showing PAni particle dispersion in ethanol-water (50%;50%) stripped of the electrode (upper left) and SEM images of the same when particles are separated by using a voltage ≈2.0 V.

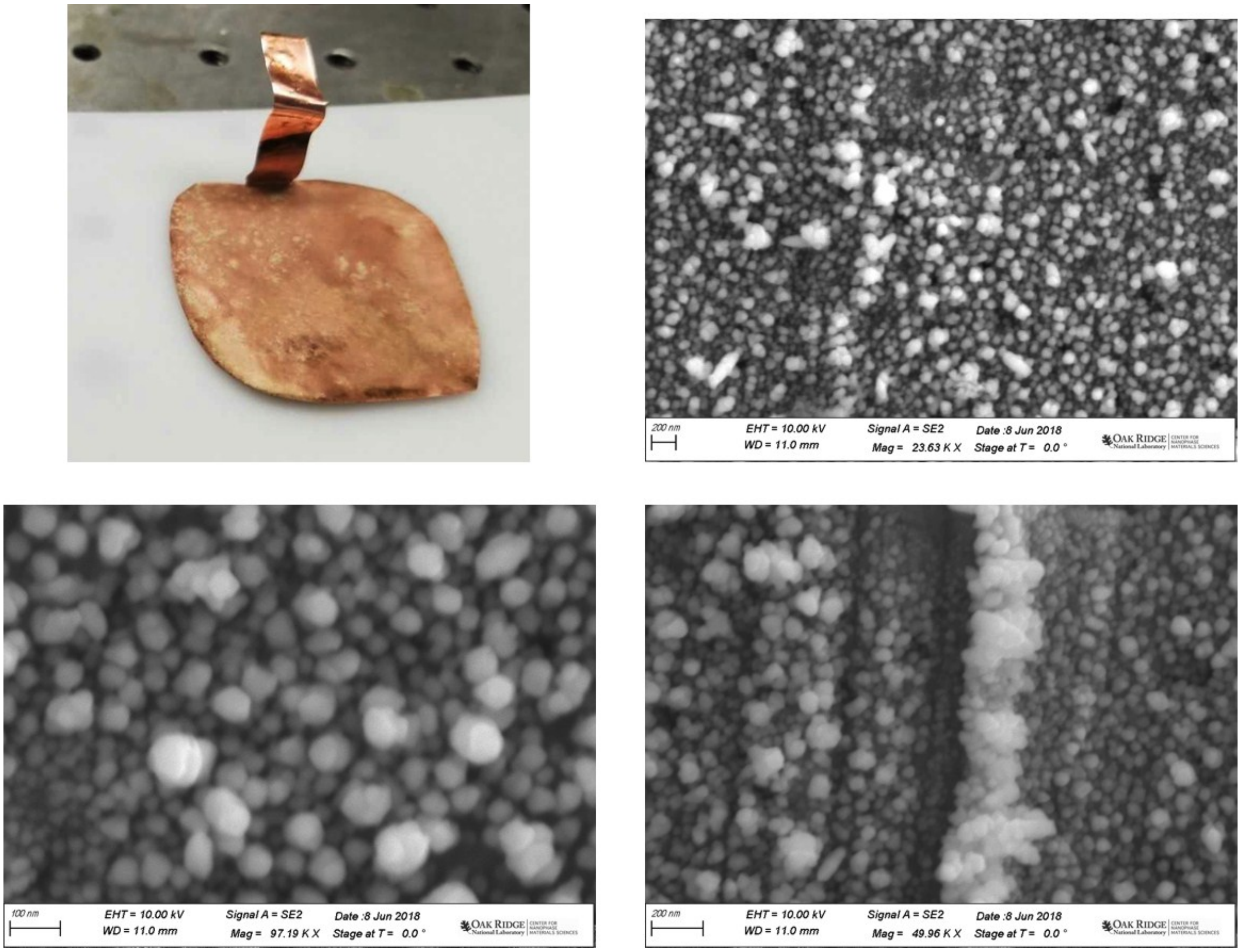

Figure S3. Showing photo of negative electrode having particles on it (upper left) and SEM images of the same electrode when particles are separated by using a voltage ≈0.6 V.

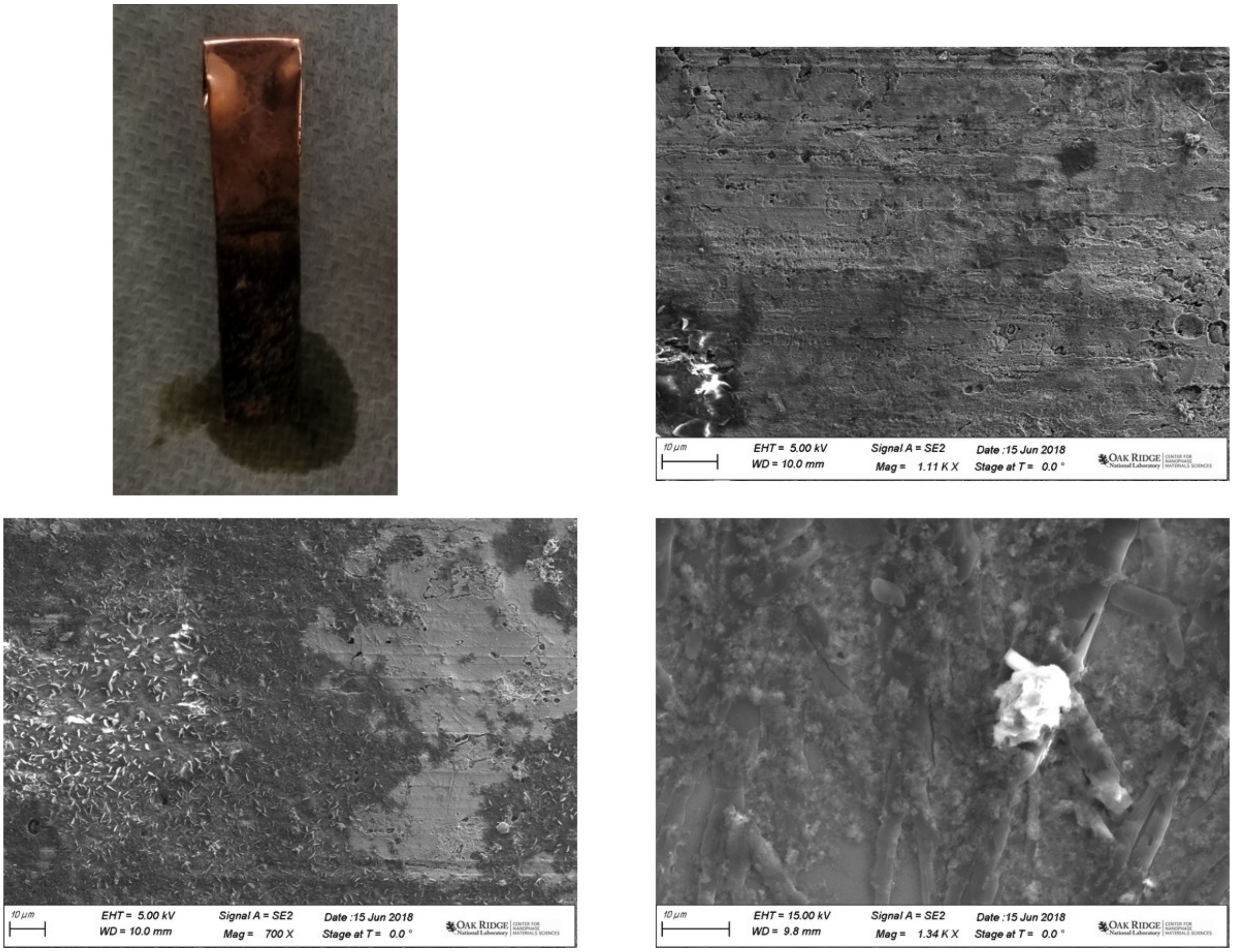

Figure S4. Showing photo of positive electrode having DBSA and other negatively charged impurities on it, (upper left) and SEM images of the same electrode when particles are separated by using a voltage ≈0.6 V.

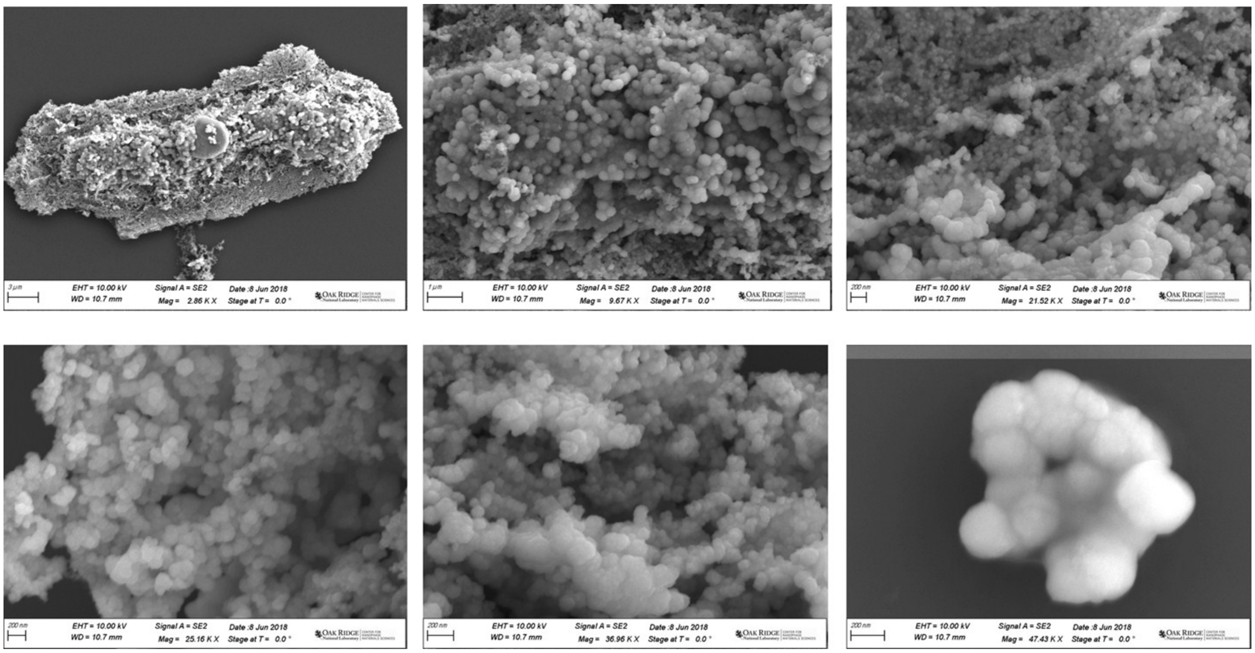

Figure S5. Some more images of PAni particles on the -negative electrode. Sometimes aggregation occurs on the surface of the electrode.

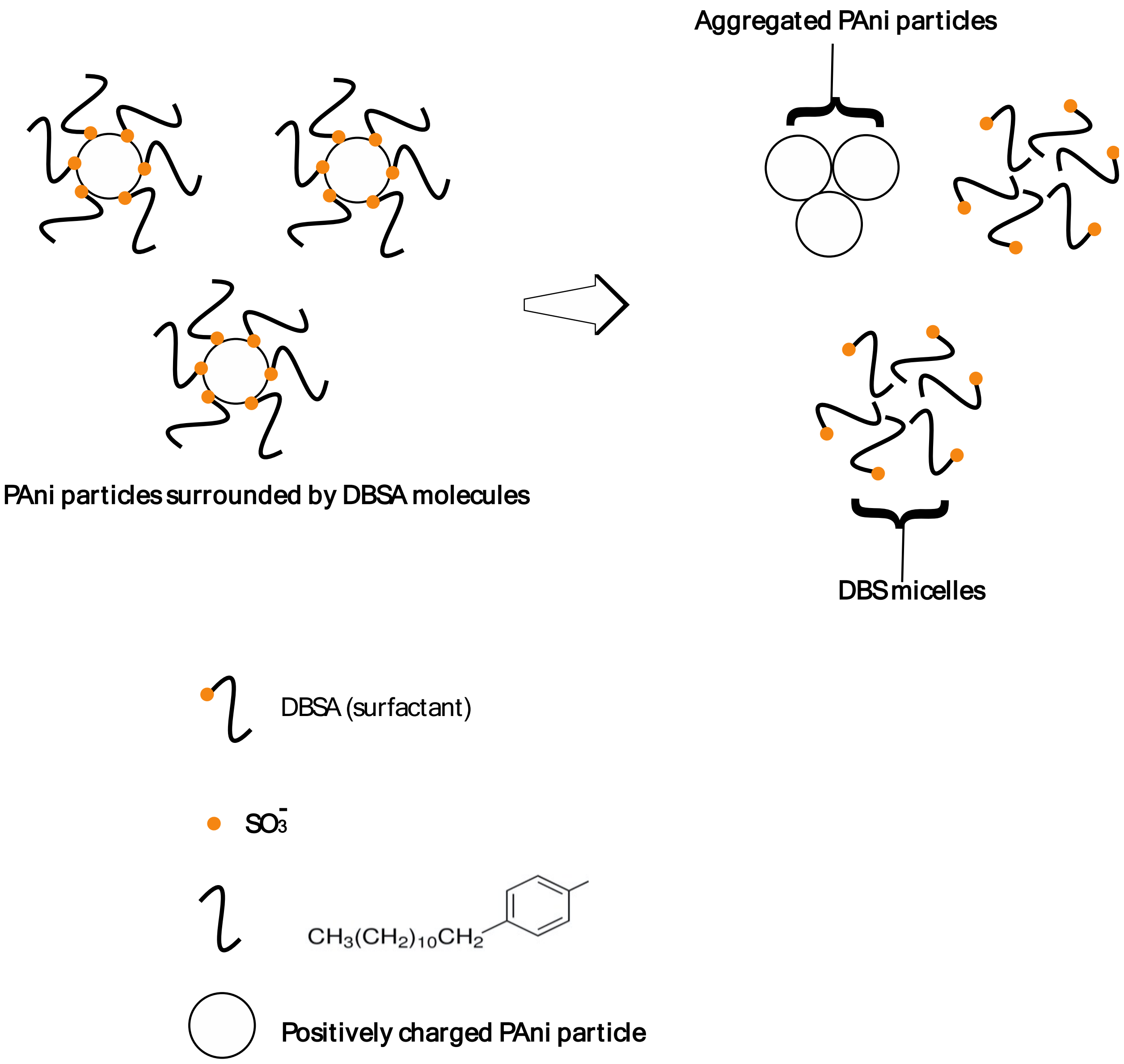

Figure S6. Assumed mechanism of PAni particle aggregation.

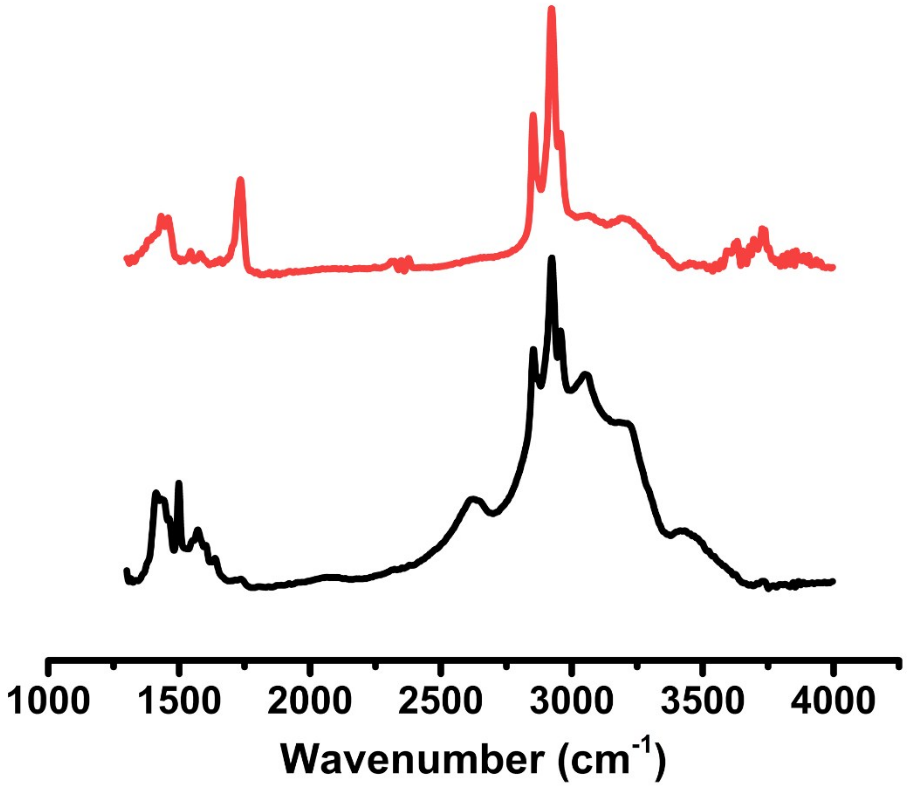

Figure 7. FTIR analysis of particles purified by electrode-based method (red line) and washing-based method (black line).
